# Case of Hydrocephalus

**Published:** 1816-08

**Authors:** David Uwins

**Affiliations:** Physician to the City Dispensary.


*For the London Medical and Physical Journal.*

*Case of Hydrocephalus;* by David Uwins, M.D. Physician to the City Dispensary.

SARAH ALDOUS, aged eight years, who resides at No. 37, Little Leonard-street, Shoreditch, became a patient of the City Dispensary on the 27th of April last. I visited her on that day, and found her lying in a comatose state, so that it was with the utmost difficulty impressions could be made upon her, and she could not be roused to any thing like notice of surrounding objects. The history which her mother gave me of her ailment was briefly as follows:

On the 5th of last March, the child complained of a violent pain over the left temple, and a pain, with stiffness of the knee, on the opposite side. These symptoms were accompanied by a very considerable irritability to the light; and, by what I could gather from the whole of the mother's narrative, all the most prominent tokens were present of incipient hydrocephalus. Her alvine evacuations, let it be observed, were at first green, and afterwards assumed a dark colour, colour, which the mother compared to the appearance of soot. A physician of eminence, in the city, first saw this child, and ordered leeches to be applied to the temples, which were followed by blisters both behind the ears and to the neck. Some medicine, too, was prescribed, which, from the manner in which it was ordered to be taken, and from the effects it produced, I judged to be composed of calomel as its main ingredient. As the parents are not in circumstances to afford much expenditure for medicine, my little patient was taken from the hands of the gentleman just alluded to, and placed under the care of another physician of eminence in one of the Dispensaries, whose mode of treating the complaint was but a continuation, as far as I could learn, of the original plan,—blistering, and the administration of calomel powders. As, however, the residence of the child was not within the visiting limits of this charity, her mother procured a letter of recommendation to the City Dispensary; and it was nearly eight weeks from her first complaining, that I visited her for the first time. The symptoms have been above described: she was in a state of sopor, and there was a hectic flush in the cheek, and a seeming state of paralytic weakness.

As the calomel plan had been persisted in for some weeks with no apparent advantage, and as there was certainly some obscurity with regard to the nature of the disorder, I judged it expedient to try, for some little time, the tonic mode of treatment, and prescribed a powder, to be taken twice a-day, composed of ten grains of Peruvian bark, and ten grains of valerian. In addition to this, I ordered five drops of the College tincture of fox-glove night and morning. In about a week's time from its commencement, I laid aside the use of the powder, determining to trust the case entirely to fox-glove and mercury in small quantities. Accordingly I directed five grains of the Pil. Hydrargyri to be divided into three doses, one of which was given every night, and the drops, now increased to six, were administered regularly night and morning; and this method of treatment has been persisted in to this day, which is about seven weeks from its commencement, with an occasional but very sparing employment of a calomel purge,—the drops eventually being augmented to eight.

Until the beginning of last week, the comatose state continued so obstinate, the child pertinaciously refusing almost every thing but her medicine, that I must confess I calculated upon it with some certainty as a lost case; and had so far abandoned all hopes of recovery, as to have spoken to one of my colleagues to request his attendance and assistance at at the examination which I proposed to make of the encephalon and viscera, as soon as the fatal termination should be made known to us. It was, as I have stated, a little more than a week since that the mother came to me at the Dispensary, and told me that the child was getting better; that they could rouse her, that she spoke to them, and would even stand on her legs. And now, for the first time, (I wish the reader to observe this,) did the mother perceive a little affection of the mouth, as if the constitution had just begun to acknowledge visibly the influence of mercury. From this period she has been rapidly improving, and yesterday, when I called at the house, she came running to the door, full of animation, and free from complaint.

*Observations.*—There are three points to which I will take the liberty of soliciting the reader's attention in reference to the peculiarities and bearings of the above narration.

In the first place, the two prominent and primarily observed symptoms, namely, the pain in the temple and the affection of the knee on the opposite side. I do not mean at all to enter upon the pathology of these concomitant tokens of brainular mischief, further than to point out the almost invariable connection they have the one with the other; and I am the more anxious to insist upon the weakness of the knee (or tripping of the foot, which is from the same source) as being, in almost all instances, the very first sign of hydrocephalic irritation, both because I think the symptom has been by far too little recognized by practitioners and authors, and because it has become too much the fashion to look no further for either cause or symptoms than the alimentary and intestinal canal. Let me not be understood to undervalue, in any measure, the importance of minutely noticing the state of the alvine excretions; but do let us recollect that there are such things as brain and nerves, as well as stomach and bowels. Now, by whatever bond of connection that inflammatory or irritative action of the brain, which marks the first stage of hydrocephalus, produces the lameness or tripping just noticed, certain it is, that, in a more or less defined degree, the thing itself, by proper scrutiny, will be almost invariably detected. I speak, of course, from my own observation, and, as it is of much moment for the practitioner to lay hold early of a pathognomic sign of this insidious disease, I have judged it right, in this place, thus to particularize it.

The second circumstance to which I would request attention, as it respects the case before us, is the order and manner in which the stomach and bowels became affected. "At first the stools were green, and afterwards dark," according to the mother's account, which would seem to argue, that the irritation in the biliary organs was the immediate consequence, not the cause of the derangement in the head; and that the subsequent torpor of the secretions, and darkness of the stools, thereby occasioned, was the result of the continued and unmitigated violence of the primary disorder. The calomel, too, it should seem, was, in reference to the radical state of affairs, entirely thrown away, while its action was not mercurial, but merely purgative; and this brings me to the last particular to be noticed, namely, the apparent cessation of the complaint as connected with the commencement of the mercurial action. What precise effect the mercury produced it is not easy to say, but I think the whole history of the case pretty fairly establishes the assumption, that it was the excitation of a new action in some part of the lymphatic or secretory organs that cured the complaint. It would appear that in all cases of affections of the brain, especially when, as in the present instance, they put on somewhat of a chronic character, the gradual insinuation of mercury into the habit is that mode of administering this remedy which has the best chance of proving beneficial. Had this child been, in the first instance, salivated, it is, I think, very probable that the powers of the system would have sunk before the malady was subdued.

With respect to the share that the digitalis might have had in bringing about the salutary alteration, I am well aware that a great many will be sceptical; but, in my opinion, this very powerful medicine is unduly appreciated by those who confine their views to its sedative effects as a counteractive of inflammation. Like mercury, when given in small and gradually augmented quantities, I have witnessed its influence upon the lymphatic system with as much clearness and satisfaction as can be obtained in the instance of any medicine whose operation is not immediately perceptible. In *tabes mesenterica*, for example, fox-glove has been administered, under my direction, in extremely small and perseveringly increased doses, with the most unequivocal and happy results. But on this subject I will not now enlarge. I may probably, in a short time, again venture to solicit for a few pages of the Medical and Physical Journal, in order to put together some further hints on the subject of medicinal operation upon the lymphatic system in young persons; on the connection of tabes mesenterica with chronic hydrocephalus; and on the comparative virtues, as well as combined effects, of mercury, fox-glove, and steel, in several disorders incident to children.

*Thavies Inn; June* 25, 1816.

---

We are much obliged to Dr. Uwins for the above, and shall thankfully receive his other practical remarks.—Edit.

